# Learning strategies of undergraduate nursing students during the COVID-19 pandemic

**DOI:** 10.1590/0034-7167-2022-0764

**Published:** 2023-10-06

**Authors:** Ana Carolina Bezerra de Lima, Danielle Christine Moura dos Santos

**Affiliations:** I Universidade de Pernambuco. Recife, Pernambuco, Brazil

**Keywords:** Learning, Students, Nursing, Education, Distance, SARS-CoV-2, Cross-Sectional Studies, Aprendizaje, Estudiantes de Enfermería, Educación a Distancia, COVID-19, Estudios Transversales, Aprendizagem, Estudantes de Enfermagem, Educação à, Distância, COVID-19, Estudos Transversais

## Abstract

**Objectives:**

to analyze the learning strategies used by nursing students from a public university, in remote teaching, during the COVID-19 pandemic.

**Methods:**

a cross-sectional study developed with nursing students who attended remote teaching classes. The sample was obtained by convenience and data were collected online, with 112 participants. Student and Mann-Whitney tests were performed for data analysis.

**Results:**

the most frequently used learning strategies were self-regulatory and cognitive. There was moderate use of interpersonal help-seeking and emotional control strategies. Male students, with a private study environment and good internet connection, used emotional control strategies more frequently.

**Conclusions:**

self-regulatory and cognitive learning strategies, appropriate for higher education, were frequently used by nursing students, which is an important tool for adapting these students to the university context.

## RESUMO

**Objetivos:**

analisar as estratégias de aprendizagem utilizadas por graduandos de enfermagem de uma universidade pública, no ensino remoto, durante a pandemia da COVID-19.

**Métodos:**

estudo transversal, desenvolvido com graduandos de enfermagem que cursaram as aulas no ensino remoto. A amostra foi obtida por conveniência, e os dados foram coletados de forma *online* com 112 participantes. Realizaram-se os Testes t de *Student* e Mann-Whitney para análise dos dados.

**Resultados:**

as estratégias de aprendizagem utilizadas com mais frequência foram as autorregulatórias e cognitivas. Houve uso moderado das estratégias de busca de ajuda interpessoal e de controle emocional. Estudantes do sexo masculino, com ambiente de estudo privativo e boa conexão com a internet, utilizaram mais frequentemente estratégias de controle emocional.

**Conclusões:**

estratégias de aprendizagem autorregulatórias e cognitivas, apropriadas ao Ensino superior, foram frequentemente utilizadas pelos graduandos de enfermagem, o que se configura importante ferramenta para adaptação desses estudantes ao contexto universitário.

## RESUMEN

**Objetivos:**

analizar las estrategias de aprendizaje utilizadas por estudiantes de enfermería de una universidad pública, en la docencia a distancia, durante la pandemia del COVID-19.

**Métodos:**

estudio transversal desarrollado con estudiantes de enfermería que asistieron a clases de enseñanza a distancia. La muestra se obtuvo por conveniencia, y los datos se recolectaron en línea, con 112 participantes. Se realizaron las pruebas t de *Student* y Mann-Whitney para el análisis de datos.

**Resultados:**

las estrategias de aprendizaje más utilizadas fueron la autorregulatoria y la cognitiva. Hubo un uso moderado de estrategias de búsqueda de ayuda interpersonal y control emocional. Los estudiantes varones, con un ambiente de estudio privado y buena conexión a internet, utilizaron con mayor frecuencia estrategias de control emocional.

**Conclusiones:**

las estrategias de autorregulación y aprendizaje cognitivo, propias de la educación superior, fueron utilizadas con frecuencia por los estudiantes de enfermería, lo que constituye una herramienta importante para la adaptación de estos estudiantes al contexto universitario.

## INTRODUCTION

With the declaration of the COVID-19 pandemic, caused by the Severe Acute Respiratory Syndrome Coronavirus 2 (SARS-CoV-2), health authorities around the world established guidelines to mitigate the spread of the virus, including the interruption of teaching activities present in educational institutions^(1-2)^. In this context, educational institutions began to temporarily adopt activities developed through remote teaching, with the replacement of face-to-face classes for pedagogical activities mediated by digital technologies^(3)^.

The learning developed in the remote environment, also called online or virtual learning, allows students to access knowledge in a non-linear way, whose connections between individuals and the environment are established from variable but interconnected places^(4)^. The environment, from the digital perspective, is constituted by cyberspace, an environment for open communication, globally interconnected by the internet^(5)^. Thus, the digital space comes to be understood as a learning space, guaranteed by means of tools that do not require the simultaneous presence of those involved to establish communication^(4)^.

In remote teaching, students’ autonomy is one of the most striking characteristics, as students will be able to choose and manage, among other factors, the learning strategies that they will use to achieve certain tasks and established goals^(6-7)^. Learning strategies are procedures that students intentionally use to learn a subject or perform a certain activity, which can be adapted to each type of activity or environment to increase learning effectiveness^(8)^. Therefore, in courses held remotely, using learning strategies appropriate to this environment can guide the planning of these courses and improve student achievement^(9)^.

Among the main learning strategies, cognitive strategies stand out, related to individuals’ executive functions, such as reading, taking notes, summarizing, asking questions and others, and the metacognitive or self-regulatory strategies, related to learning planning, monitoring and assessment^(6)^. It is necessary to understand the learning strategies that students use to study in diverse settings, such as university education and remote learning so that teaching methodologies can be planned by educational institutions, according to the profile of learners^(7)^.

In a scoping review of the learning strategies used in remote teaching during the COVID-19 pandemic, it was noticed that studies were concentrated in developed and high-income countries, which demonstrates the need to carry out further investigations in middle-income countries and low income^(10)^. Another striking characteristic, highlighted in the literature, was the lack of studies that identify the learning strategies specifically used by undergraduate nursing students, in which there is a concentration of studies published in the medical field^(11-12)^.

When considering the impacts of the COVID-19 pandemic on the quality of higher education in health, recognizing the learning strategies that nursing students are using to process information, recover prior knowledge and acquire knowledge is essential to reduce educational losses arising from the pandemic and to promote active and student-centered learning^(13)^, which highlights the pertinence of carrying out this research.

Therefore, the analysis of the learning strategies used by nursing students, as proposed in this study, allows the adoption of initiatives to help them in the training process, in addition to providing the development of educational methods consistent with education in the remote environment, improving academic performance, favoring the fulfillment of learning objectives and providing professors with the necessary tools in the educational process mediated by digital technologies^(14-15)^.

## OBJECTIVES

To analyze the learning strategies used by undergraduate nursing students from a public higher education institution, in remote teaching, during the COVID-19 pandemic.

## METHODS

### Ethical aspects

This study met the scientific requirements of research involving human beings, having complied with the norms of Resolution 466/12 of the Brazilian National Health Council (CNS)^(16)^. The main project was assessed and approved by the Research Ethics Committee of the *Hospital Universitário Oswaldo Cruz* (HUOC) Hospital Complex. Data collection followed the guidelines for research procedures with any stage in a virtual environment, published by the Brazilian National Research Ethics Committee (CONEP)^(17)^. The research participants completed the Informed Consent Form (ICF).

### Study design, place and period

This is a cross-sectional, exploratory and quantitative study, related to a macroproject entitled “*Formação de Graduandos e Pós-graduandos em Enfermagem em Tempos de Pandemia do Coronavírus SARS-COV-2*”, linked to the Associated Graduate Program in Nursing at the *Universidade de Pernambuco/Universidade Estadual da Paraíba* (PAPGEnf-UPE/UEPB). The manuscript was guided by the STrengthening the Reporting of OBservational studies in Epidemiology (STROBE) framework, which provides guidelines for reporting observational studies in epidemiology^(18)^.

The study was carried out at the *Faculdade de Enfermagem Nossa Senhora das Graças* (FENSG), at the *Universidade de Pernambuco* (UPE), from September 2020 to December 2021, when the educational institution had authorized the replacement of face-to-face classes with mediated classes. through remote teaching, in compliance with health requirements for managing the COVID-19 pandemic.

### Population, sample and selection criteria

The study population consisted of 395 nursing students from curricular modules I to VIII who attended classes through remote teaching, where each curricular module corresponds to a school semester at the institution. The sample was obtained non-probabilistically, for convenience, by calculating the sample size for frequency in a population, using a 95% confidence interval and a 5% margin of error, totaling a sample of 195 participants^(19)^. Nursing undergraduates enrolled in the supplementary term 2020.3 and/or academic semester 2020.1, aged 18 years and older, were included. Students who had not completed the additional period 2020.3 and/or academic semester 2020.1 (enrollment suspension, medical leave, gestational leave or other reasons) were excluded.

### Study protocol

To recruit participants, the course coordination was asked to provide a list of eligible students, with their respective telephone contacts and e-mail addresses. The form with the measurement instruments was structured in Google Forms^®^ and sent via email to eligible students. The invitation to participate in the research, the ICF and the research instruments were part of this form, namely the Sociodemographic Questionnaire, prepared by the research team, and the Learning Strategies Scale (EEA), by Martins and Zerbini (2014)^(9)^.

The individual questionnaire for participant sociodemographic and digital characterization was prepared by the researchers for exclusive use in this study. Its construction was based on literature on the transformations that occurred in educational institutions in the face of the implementation of remote teaching in the COVID-19 pandemic and on the importance of characterizing the digital profile of students who were receiving classes in this format^(14-15,20-25)^.

To assess the learning strategies used by nursing students, the EEA by Martins and Zerbini (2014) was adopted, adapted and validated for use in Brazil, specifically in distance higher education activities. The scale consists of 29 items, which describe the behaviors used by students to study. These items are associated with an 11-point Likert-type scale (ranging from zero to 10 points, where 0 = never and 10 = always). The EEA has a structure composed of four factors: cognitive strategies (15 items), emotion control (4 items), self-regulatory strategies (7 items) and interpersonal help-seeking (3 items)^(9)^.

To obtain the score of the EEA factors, the arithmetic mean of the values indicated by the respondents in the items of each factor must be calculated. As EEA ranges from zero to 11 points and, therefore, the items are positive, the greater the mean obtained in each of the factors, the greater the frequency with which students use the learning strategies for that factor. Mean values between zero and 4.0 indicate low frequency of use; between 4.1 and 7.0, moderate use; between 7.1 and 10, frequent use^(9)^.

Before starting data collection, a pilot test was carried out using a form containing the ICF and the research instruments, sent to a group of students through the same process with which it would be forwarded to the other participants so that it could undergo an assessment regarding term clarity and precision, instruction objectivity, ICF understanding, instrument adequacy to the digital form, form layout, ease of filling in the data, need for adaptations and other aspects. The observations scored in this test were recorded by the researchers, through feedback from students by email, telephone contact and videoconference, using Google Meet^®^. Only after adapting the pilot test, data collection began, which took place between July and December 2021. The number of students in the pilot test was not added to the final sample.

### Analysis of results, and statistics

Software R-project, version 3.4.2, was used to analyze the data. The Kolmogorov-Smirnov test was applied to assess data distribution, considering p-value < 0.05 to reject the hypothesis that data are normally distributed. Categorical variables were expressed through absolute and relative frequencies. Numerical variables were expressed as mean ± standard deviation (Min-Max). For comparison between two groups, Student’s t test or the Mann-Whitney test were used.

## RESULTS

A total of 112 nursing students participated in the study, with a predominance of women (89.3%), aged between 18 and 28 years (mean of 20.9 ± 2.1 years), white color/race (52.7%), single (95.5%), without comorbidities (87.4%), with monthly family income of up to three minimum wages (60.7%), from urban areas (98.2%), who lived in their own house (75.0%) and shared housing with other people (98.2%). There was a prevalence of nursing students in curricular module II (23.2%), in module I (19.6%) and in module V (15.2%), respectively.

According to their education, 61.6% of them were fully trained in a private school: 16.1% mostly in a private school; 13.4% fully in public school; and 8.9% mostly in public school. As undergraduates were attending classes through remote teaching, it was seen that 81.3% of students considered having a good quality Internet connection and 52.7% of them declared studying in a private environment.

Regarding the results of applying the LES by Martins and Zerbini (2014) to nursing undergraduates during remote teaching, values were initially presented according to each of the four domains (Table 1).

**Table 1:** Descriptive results of the Learning Strategies Scale by Martins and Zerbini (2014), according to domains (N = 112), Recife, Pernambuco, Brazil, 2022

Learning Strategies Scale (EEA) domains	Mean	Median	Standard deviation	Minimum value	Maximum value	*p* value^*^
Self-regulatory strategies	7.4	7.3	1.7	3.0	10.0	0.869
Cognitive strategies	7.2	7.4	1.4	1.3	9.9	0.883
Interpersonal help-seeking	6.2	5.1	2.1	1.7	10.0	0.727
Emotion control	5.7	5.7	2.0	1.5	10.0	0.999

* p values from the Kolmogorov-Smirnov normality test.

On average, undergraduates showed frequent use of self-regulatory and cognitive learning strategies as well as moderate use of interpersonal help-seeking strategies and emotion control. Moreover, data relating to the scores of each of the learning strategies used by students were also analyzed (Table 2), whose percentage of concentration of responses indicates the frequency with which each strategy was used.

**Table 2:** Descriptive analysis of the Learning Strategies Scale by Martins and Zerbini (2014), according to items (N = 112), Recife, Pernambuco, Brazil, 2022

Learning Strategies Scale Items	Mean	Median	Standard deviation	Min	Max	*p* value^*^	Frequency of use (%)		
							0-4.0	4.1-7.0	7.1-10
1. I kept calm when I had difficulties	6.0	6.0	2.2	0	10	0.064	20.5	52.7	26.8
2. I repeated to myself, when I felt anxious, that everything would be fine at the end of the course	6.9	7.0	2.7	0	10	0.020	20.5	31.3	48.2
3. I kept calm with the possibility of having a below-expected income	4.4	4.0	2.5	0	10	0.013	58.0	29.5	12.5
4. I remained calm in the face of the mistakes I made when carrying out course activities	5.4	5.4	5.0	1	10	0.183	36.6	41.1	22.3
5. I tried harder when I realized I was losing concentration	7.3	8.0	2.1	0	10	0.001	10.7	37.5	51.8
6. I forced myself to keep my attention on my studies when I felt uninterested	6.9	7.0	2.3	0	10	0.003	16.1	34.8	49.1
7. I tried harder when I realized I was losing interest in the subject	6.7	7.0	2.2	0	10	0.016	15.2	42.0	42.9
8. I revised the material to verify how much I mastered the content	6.8	7.0	2.7	0	10	0.001	20.5	31.3	48.2
9. I made an effort to check my understanding of what was being taught	7.6	8.0	2.0	2	10	0.002	6.3	34.8	58.9
10. I tried to solve my doubts by consulting the course support materials	8.1	9.0	2.0	1	10	0.000	5.4	26.8	67.9
11. I tried to better understand the contents by studying them in the course material	8.1	8.5	1.9	0	10	0.000	2.7	24.1	73.2
12. I sought help from the professor or monitor to clarify my doubts about the content	5.1	5.5	3.0	0	10	0.034	35.7	42.9	21.4
13. I sought help from colleagues to clarify my doubts	4.8	5.5	3.7	0	10	0.000	42.9	24.1	33.0
14. I exchanged messages with colleagues to clarify doubts about the course content	8.8	10.0	1.9	0	10	0.280	3.6	13.4	83.0
15. I looked for other sources of research, outside the internet, related to the course to help me learn	7.2	8.0	3.2	0	10	0.000	18.8	20.5	60.7
16. I looked for websites related to the course content to help me learn	9.0	10.0	1.6	0	10	0.296	1.8	11.6	86.6
17. I tried to understand the content by applying it in practice, instead of reading it or asking someone for help	5.2	5.0	2.8	0	10	0.015	42.9	32.1	25.0
18. I revised the contents related to the exercises in which I made mistakes	6.3	7.0	2.8	0	10	0.000	24.1	26.8	49.1
19. I learned content by mentalizing them repeatedly until I realized that I had understood	6.5	7.0	2.8	0	10	0.013	23.2	33.0	43.8
20. I mentally repeated the course contents that I would like to learn	6.6	7.0	2.9	0	10	0.003	20.5	33.0	46.4
21. I took notes on course content	8.4	9.0	2.1	0	10	0.000	6.3	17.0	76.8
22. I made summaries of course content	7.6	9.0	2.8	0	10	0.000	14.3	17.9	67.9
23. I read the course content several times	6.6	7.0	2.8	0	10	0.017	20.5	35.7	43.8
24. I made outlines of the course content	6.4	7.0	3.0	0	10	0.002	25.0	27.7	47.3
25. I reflected on the implications of the contents learned	7.4	8.0	2.3	0	10	0.000	11.6	28.6	59.8
26. I tried to develop a global idea about how the course contents related to each other	7.4	8.0	2.3	0	10	0.002	11.6	31.3	57.1
27. I associated the course contents with my previous knowledge	8.4	9.0	1.6	3	10	0.002	2.7	9.6	77.7
28. When analyzing the course contents, I differentiated the most important aspects from the least important ones	7.7	8.0	1.9	0	10	0.000	8.0	33.9	58.1
29. I identified daily situations in which I could apply the course contents	8.0	8.0	2.0	0	10	0.001	6.2	26.8	67.0

* p values from the Kolmogorov-Smirnov Normality Test. T.N.: this scale has been freely translated, as it does not have an English version, only Portuguese.

Finally, analyzes were carried out between the sociodemographic and digital variables of nursing students and the EEA domains, whose statistically significant associations are described in Table 3.

**Table 3:** Associations between the Learning Strategies Scale by Martins and Zerbini (2014) and socioeconomic and digital variables (N=112), Recife, Pernambuco, Brazil, 2022

Learning Strategies Scale	Sociodemographic and digital variables	Mean ± standard deviation (Min-Max)	*p* value
Sex			
Emotion control	Female	5.6 ± 2.0 (1.5 - 10)	0.017^*^
	Male	6.7 ± 1.3 (5 - 9)	
Self-regulatory strategies	Female	7.4 ± 1.7 (3 - 10)	0.117^**^
	Male	6.9 ± 1.2 (5.3 - 8.6)	
Interpersonal help-seeking	Female	6.3 ± 2.1 (1.7 - 10)	0.725^*^
	Male	6.1 ± 1.6 (3.3 - 8.3)	
Cognitive strategies	Female	7.3 ± 1.5 (1.3 - 9.9)	0.259^**^
	Male	6.9 ± 1.4 (3.7 - 8.7)	
Study environment with privacy to attend remote classes			
Emotion control	No	5.2 ± 0.3 (1.5 - 9)	0.012^*^
	Yes	6.1 ± 0.2 (2 - 10)	
Self-regulatory strategies	No	7.1 ± 0.2 (3 - 9.7)	0.156^**^
	Yes	7.6 ± 0.2 (3.6 - 10)	
Interpersonal help-seeking	No	6.5 ± 0.3 (1.7 - 10)	0.241^*^
	Yes	6 ± 0.3 (1.7 - 10)	
Cognitive strategies	No	7.2 ± 0.2 (3.7 - 9.9)	0.940^**^
	Yes	7.3 ± 0.2 (1.3 - 9.8)	
Good internet connection quality to attend remote classes			
Emotion control	No	4.8 ± 0.4 (1.5 - 9)	0.023^***^
	Yes	5.9 ± 0.2 (2 - 10)	
Self-regulatory strategies	No	7.3 ± 0.3 (4.6 - 9.3)	0.636^****^
	Yes	7.4 ± 0.2 (3 - 10)	
Interpersonal help-seeking	No	6.4 ± 0.4 (3 - 10)	0.700^***^
	Yes	6.2 ± 0.2 (1.7 - 10)	
Cognitive strategies	No	7.3 ± 0.3 (3.7 - 8.8)	0.636^****^
	Yes	7.2 ± 0.2 (1.3 - 9.9)	

Note: Min - Minimum value; Max - Maximum value;

* Student’s t test;

** Mann-Whitney test.

## DISCUSSION

Learning strategies indicate part of students’ individual characteristics in relation to learning, considering that this may be associated with individuals’ experiences and their interaction with the environment that surrounds them^(7)^. In view of this, research on the study of learning strategies was identified as one of the factors related to the adaptation of undergraduate students, providing important elements on how academic learning occurs^(26)^.

The present study identified that undergraduate nursing students, in remote teaching, made frequent use of self-regulatory learning strategies. Strategies stand out in research on how university students’ study, and this is associated with the fact that students use them to monitor what is being learned and to reflect on learning quality^(9,27)^. From the perspective of virtual learning, it was noticed that students who adopted self-regulatory strategies obtained better averages in formal assessments, helping them in the process of self-management of learning^(28)^.

Corroborating this study, self-regulatory strategies were prevalent in investigations that assessed the learning strategies used by undergraduate students. In the study that analyzed the strategies used by university students in a hybrid learning environment, it was evidenced that the use of self-regulatory strategies was related to the improvement of academic performance, the encouragement of student motivation, the improvement of study habits and the choice of adequate resources for learning^(29)^.

A longitudinal study, which investigated the impact of self-regulated learning during the COVID-19 pandemic, showed that 60% to 85.8% of assessed students were able, through the use of self-regulatory strategies, to structure everyday learning in the home environment and manage the academic tasks assigned to them. The favorable result was associated with the self-organization capacity, academic performance and motivation for learning that students had before social distancing and, consequently, learning gains during remote teaching^(30)^.

In addition to self-regulatory strategies, the results of this study showed that cognitive learning strategies were also frequently used by the assessed students. A similar result was found in another investigation that sought to understand the predictive effects of learning strategies on the academic performance of university students in distance education activities, whose cognitive strategies achieved prominence, and their use was considered a positive predictor of academic performance^(31)^.

Cognitive strategies help students work directly with information, relating it to basic learning processes, such as processing, storing and retrieving information^(32)^. A study that sought to formulate recommendations for the development of remote learning in the COVID-19 pandemic proposed a work model to be developed in higher education that considered cognitive aspects (internal factors) and their association with emotional, motivational and social aspects (external factors). In this model, cognitive aspects proved to be important for learners to select, organize and integrate relevant information in the remote environment^(33)^.

In the present study, the moderate use of interpersonal help-seeking strategies was identified, unlike a study that investigated the use of learning strategies in a university course conducted at a distance, in which it was shown that interpersonal help-seeking strategies were among the most used. The study also concluded that these strategies enhance social adaptation, i.e., the establishment of relationships with those involved in the learning process (students, professors and educational institutions), since students seek on their own initiative, help from other people (tutors or classmates) to resolve doubts, thus establishing interpersonal relationships^(26)^.

In bibliometric research on learning strategies, interpersonal help-seeking was the strategy that most stood out in research in which the focus was on understanding individual learning, especially in public schools^(27)^. Interpersonal help-seeking was also identified as an effective tool in teaching in virtual learning environments, in a study that discussed aspects of student engagement in technology-mediated education, having been identified that the collaborative interaction with colleagues and professors, building effective relationships between peers, increases the level of confidence of students and enables deeper learning in activities that use digital tools^(34)^.

Emotion control strategies were also used moderately by the participants of this research. This result was similar to that found in research with university students from a distance course, in which it was noticed that, when going through adverse situations, students may not deal well with these situations, using emotion control strategies less frequently^(32)^. Emotional control has important functions in terms of learning, as emotions are linked to processes such as awareness, judgment and reasoning, in addition to influencing personality and awareness evolution, i.e., the process of human development^(35)^.

It was verified, in the present investigation, that the search for sites related to the course content, the association of the course subjects with students’ previous knowledge and note taking on these subjects, in addition to message exchange with colleagues, were individually the most used learning strategies. Similar data were found in research carried out with university students, during remote learning, whose predominant learning strategies were reading texts and preparing summaries, followed by listening to explanations about certain subjects and watching videos about them^(36)^.

On the other hand, the learning strategy least used by the students assessed here was to keep calm with the possibility of low academic results. In this regard, positive or negative emotional experiences have significant impacts on the way students face everyday challenges and, consequently, interfere with performance during online academic activities, as emphasized in a study that aimed to analyze emotions, coping strategies and self-regulated learning of students, during home isolation, due to COVID-19^(37)^. Thus, remaining calm or not in the face of possible mistakes or probable lower-than-expected academic performance may indicate the difficulty of students in dealing with emotional issues during a higher education course offered at a distance^(32)^.

Associations between gender and the use of learning strategies showed, in the present study, that male students had greater emotional control than female students. This is consistent with what was presented in the study that analyzed the use of learning strategies in a distance university course, in which male participants obtained higher scores in the “emotion control” factor, suggesting that this group managed to remain calm more easily in the face of the difficulties encountered in the course, compared to the group of female students. This finding was related to the fact that male students organize, according to the authors, the time and place of study in a more disciplined way, relevant variables when considering the use of learning strategies^(26)^.

Also, it was evidenced, in this research, that the quality of internet access influenced the learning strategies that nursing students most adopted, in which those who considered to have a good quality internet connection used emotion strategies more frequently control. This may be related to what was presented in a study that explored emotional regulation of nursing students during the COVID-19 pandemic, in which it was emphasized that, in populations of predominantly young university students, the ability to regulate emotions is still under development and, therefore, the way of controlling emotions can be impaired in contexts of difficulties^(38)^.

In the present study, nursing students who considered studying in a private study environment more frequently used learning strategies related to emotional control. It is noteworthy that these graduates witnessed sudden changes in the various social activities required, due to the COVID-19 pandemic, in which coping measures, such as home isolation, quarantine, restriction of movement of people, distance from face-to-face workplaces, closure of educational institutions and others, were adopted, leading individuals to stay longer than usual at home, whether carrying out work activities in the home office mode, carrying out educational activities in remote teaching or even in compliance with restrictive measures^(1-2,11,13)^.

Considering this scenario, new challenges were encountered by individuals who had to study at home during the COVID-19 pandemic, often sharing a home with several people, including the need to restructure the study environment^(21,24)^. In this sense, the adequate space for studies was pointed out as a positive element for managing emotions and increasing student motivation in online subjects, thus contributing to learning^(36)^. In the home environment, study time seems to be longer, and space is often shared with other family members, which requires students to manage study goals, restructure daily tasks and control possible distractions, while it is also important for family members to understand this new configuration^(39)^.

A study identified that home isolation, during the COVID-19 pandemic, caused changes in the learning strategies used by nursing students, in which changes in the ability to manage time according to demands, self-discipline, concentration and resolution of doubts with colleagues or professors were necessary for continuity of training^(40)^. The relationship between these findings and what was found in the present study can be seen, as participants sought to resolve doubts with classmates and professors during classes in the remote format as well as the search for concentration during the course of their studies. Other impacts caused on the learning behaviors of university students during the pandemic were the deterioration in academic performance, study time and concentration, variables associated with the interference of social isolation in these individuals’ mental well-being^(11)^.

In view of this, the social distance necessary to face the COVID-19 pandemic served as a basis for strengthening learning in remote teaching, enabling the construction of new paradigms for the construction of knowledge and, consequently, reflecting on the need to find learning strategies suited to these paradigms^(14,22)^. The literature shows that identifying the learning strategies used during the pandemic makes it possible to qualify the educational process, as it makes it possible to equip professors and coordinators of educational institutions to plan pedagogical activities, with methods suitable for different learning styles^(10)^.

### Study limitations

The present study has some limitations, such as not being able to obtain the significant participation of undergraduates from the institution where the study was developed, who may not have had access to the digital data collection questionnaire, either because of internet connection instability or related to their area of residence (especially those students who lived in rural areas). In addition, personal factors such as sleep quality, physical exercise, whether or not students were affected by COVID-19, motivation for studying and others, may also have interfered with academic performance and use of learning strategies during the pandemic, but these variables were not assessed in this study.

### Contributions to nursing and health

Knowing the learning strategies that university students use to learn in the remote environment is a powerful tool for planning educational activities in higher education in health, especially in the remote teaching scenario. For nursing, the main benefit of this study refers to the innovative contribution of identifying the learning strategies used by undergraduate nursing students in the context of the COVID-19 pandemic, which makes it possible to verify whether the learning strategies used by students are suitable for the classes developed in remote teaching, to enhance the strategies used frequently and to encourage the teaching and practice of less used strategies.

## CONCLUSIONS

The analyzes of this study on learning strategies used by undergraduate nursing students in remote teaching revealed the frequent use of self-regulatory and cognitive strategies as well as the moderate use of interpersonal help-seeking and emotion control strategies appropriate for higher education and the remote environment. It was also evident that male university students had greater emotional control, compared to female students, and that the inclusion of learners in a private study environment and internet connection quality were associated with greater use of emotion control strategies. It is recommended that new studies be carried out to broaden the discussion on this topic, which is so much related to nursing education quality.

Therefore, this study provides essential elements for the implementation of educational programs consistent with target audience characteristics, contributing to the reduction of dropout rates as well as to the quality of future nurses’ training. Thus, assessing learning strategies can reorient educational policies and curriculum restructuring in the face of critical situations, such as the current pandemic and after it, enabling professors and managers of higher education institutions to propose viable and meaningful activities for learners.

## Data Availability

https://doi.org/10.17632/p5pg8zwz7r.1
